# Impact of Variability Factors on Hair Cortisol, Blood Count and Milk Production of Donkeys

**DOI:** 10.3390/ani12213009

**Published:** 2022-11-02

**Authors:** Federica Salari, Chiara Mariti, Iolanda Altomonte, Angelo Gazzano, Mina Martini

**Affiliations:** Department of Veterinary Science, Università di Pisa, Viale delle Piagge 2, 56124 Pisa, Italy

**Keywords:** dairy donkey, milk production, hair cortisol, blood parameters, lactation phase, parity, production season

## Abstract

**Simple Summary:**

The recent interest in donkey milk has led changes in donkey farm management; however, little is still known about the effect of farm management on donkey health and welfare. The measurement of hair cortisol is a new method to assess stress in animals. Furthermore hair cortisol measurement in dairy donkeys has not previously been done. In addition, only a few studies have investigated physiological ranges of donkey blood parameters. We analysed changes in milk quality, blood parameters and mane hair cortisol in relation to lactation phase, parity and season. Hair cortisol was higher in the peri-partum period while milk yield and composition and blood parameters changed according to parity or season. This study represented a first effort to better understand the biochemical processes occurring in lactating jennies, and their physiological and wellbeing status.

**Abstract:**

The increased interest in donkeys because of their milk has led to changes in their farm management. Little is known about the effect of the farming systems on donkey health and welfare. Measuring hair cortisol concentrations is an emerging method to assess stress in animals. To the best of our knowledge, no cortisol assessment has been done on dairy donkeys; similarly, only a few studies have investigated donkey haematological values. The aim of this study was to evaluate the effects of the lactation phase, parity and season on blood parameters, milk yield and quality and hair cortisol in dairy donkeys. Individual samples of milk, blood and mane hair were taken from twenty jennies at 1, 6 and 10 months after parturition. Higher values of hair cortisol were found in the first sampling, suggesting temporary stress during the peri-parturition. The parity influenced the number of blood cells, which was lower in the pluriparous jennies. The season affected milk quality and mean corpuscular haemoglobin and mean corpuscular haemoglobin concentration. The latters might represent the adaptation to the environmental conditions. This study contributes to a better understanding of the biochemical processes occurring in lactating jennies, and to their physiological and wellbeing status.

## 1. Introduction

With the advent of industrialization, the global donkey (*Equus asinus*) population declined drastically. However, in recent years there has been increased interest, especially in relation to the milk production of donkeys. Donkey milk is currently known for its particular nutritional and nutraceutical characteristics compared to the milk traditionally used for human nutrition [1] and is primarily targeted at sensitive consumers, such as children [2].

The high genetic resistance to diseases in donkeys and their proper management, together with the limited use of drug treatments, have guaranteed their welfare. The increased interest in donkey milk has led to changes in the farming management of donkeys from traditional to more organised farming systems [3], in order to guarantee safe and quantitatively sufficient milk production. However, little is known about how the new management systems and the extended lactation period in modern commercial farms affect the health and welfare of dairy donkeys. The livestock welfare is strongly connected to management. If an animal is not properly managed, it will probably be exposed to stress which can impact its behaviour and its health [4]. 

Stress strongly affects animal welfare [5] and the immediate physiological response to stress is increased activity of the sympathetic nervous system, which increases the heart rate [6], as well as the hypothalamic–pituitary–adrenal axis activity [7]. 

Increased heart rate and high glucocorticoid concentrations have been found in the plasma and saliva, which are strongly associated with acute stress [7,8]. According to Chrousos [9], short-term stress does not necessarily compromise the animal’s welfare; however, if it is prolonged, it can negatively affect the animal’s health. 

Measuring hair cortisol concentrations is an emerging method to assess long-term stress in animals. In fact, cortisol passively diffuses from circulating blood into the hair, where it gradually accumulates. Assessing hair cortisol can therefore provide information on the cortisol levels over the days preceding the sampling [10]. Measuring steroids in hair is non-invasive and the samples can be stored at room temperature. Although hair cortisol concentrations (HCCs) have been assessed in several species [8,10,11,12,13], to the best of our knowledge, no study has analysed HCCs in dairy donkeys.

Similarly, only a few studies have investigated the haematological values of donkeys and these studies do not take into account the multiple variability factors that affect the blood cell count, such as age, sex, time of sampling and the level of physical and productive activity [14,15,16,17].

The aim of this study was to evaluate how some variability factors such as lactation phase, parity and production season have an influence on blood parameters, on the quantity and quality of milk, and for the first time on the quantity of hair cortisol in dairy donkeys.

## 2. Materials and Methods

### 2.1. Animals and Sampling 

Twenty dairy jennies of autochthonous Amiata breed were selected for the study, of which five were primiparous, seven were secondiparous, and eight were pluriparous. The donkeys were bred on a farm located in central Italy (42530 52.59 N 10470 05.52 E, WGS84) and reared outdoors in a semi-intensive system. They were fed with mixed hay ad libitum and approximately 2.5 kg/day/head of the concentrate for dairy donkeys. Eight jennies gave birth in the autumn–winter and 12 in the spring–summer. 

The farm produces pasteurised milk for human consumption in accordance with the requirements of Regulation (EC) No 853/2004. During the first month of lactation, all the milk was left for the foal. Starting from 30 days after delivery (1 month), the jennies were machine-milked twice a day (at 11.00 and 15:00). The foals were separated from their mothers four hours before each milking. 

Individual samples were taken at 1, 6 and 10 months after parturition, with a total of 60 samples for each biological matrix:

− Blood: Peripheral blood samples were collected in EDTA tubes for complete blood cell count (CBC).
− Milk: Individual milk yield was measured by a lactometer connected to the milking machine. Each sample was obtained by mixing the two individual daily milkings refrigerated at 4 °C from which homogeneous samples of 100 mL of milk were collected.
− Mane hair: Individual samples of mane hair were collected using scissors from the midneck region, as close as possible to the skin. Since the donkey hair growth rate is still unknown, the segment length was based on previously reported hair growth rates in horses [18]. Based on Duran et al. [19], 2 cm hair segments were considered to contain the cortisol that had accumulated over one month. We divided each hair sample clump into two segments, starting from the extremity proximal to the root, two centimetres each (0–2 cm and 2–4 cm). The excess hair length was excluded. The mean value obtained for the 0–2 and 2–4 cm hair segments was used for statistical analysis, thus the hair cortisol levels referred to the two months preceding the collection.

All the milk and blood samples were refrigerated at 4 °C immediately after the sampling and analyzed within 24 h of collection. 

The procedure was communicated to the Ethics Committee of the University of Pisa, Italy and it received a favourable opinion with Decision N.34/2018. 

### 2.2. Milk Analysis

To determine the chemical quality of the milk, the following parameters were evaluated for each raw fresh milk sample: dry matter, fat, protein, casein, lactose, and urea content were measured by infrared analysis (MilkoScan 7 RM; Italian Foss Electric, Padova, Italy). The somatic cell count (SCC) was evaluated by the fluoro-opto-electronic method (Fossomatic Italian Foss Electric, Padova, Italy).

### 2.3. Hair Cortisol Analysis

After splitting hair samples into two segments as previously described (0–2 cm and 2–4 cm), gross contaminants (mud, stones, vegetation) were manually removed with tweezers. The hair of all animals was not washed immediately, but stored at room temperature and in the dark until the analysis.

The analyses were conducted in six phases, developed from the method used to assess cortisol concentrations in horses [19]. Each sample was weighed (200 mg), then washed three times in 40 μL methanol/mg hair (8 mL methanol/200 mg hair) for three minutes per wash, with manual shaking. After cleansing, the hair was dried overnight at room temperature under a fume hood. The cleansing in methanol was to prevent the measurement of cortisol contained in contaminants such as blood, saliva, faeces and urine, which can occur with free-range animals [20] and is also used in other species such as domestic dogs [13].

Each washed and dried sample was cut first with scissors and then finely minced with an electric clipper, in order to obtain sections less than 1 mm long. The prepared samples were then transferred to glass tubes, and stored at room temperature in the dark until the subsequent analysis. 

From each sample, 25 mg of ground hair was weighed, then methanol (500 μL) was added to the Eppendorf tube for extraction. After briefly being vortexed for 20 s, the tubes were kept sloped under orbital shaking for 24 h (orbital shaker DLAB SK-O180-E^®^, Dragon Laboratory Instruments Limited, Beijing, China).

Samples were then centrifuged for 15 minutes at 4500 rpm using a microCENTRIFUGETTE^®^4214-ALC (Kontron Instruments S.p.A., Milan, Italy). The supernatant was collected and put in 2 mL tubes, and then dried at 37 °C under a gentle nitrogen gas stream. The extraction procedure with methanol was repeated three times to ensure maximum cortisol recovery, each time adding the supernatant to vials that were left to dry. At the end of the drying phase, the tubes were closed and stored in a freezer at −20 °C.

Cortisol was quantified using a commercially available enzyme immunoassay kit, validated for direct immunoenzymatic determination of cortisol in saliva (Diametra^®^). The EIA kit for salivary cortisol has been used to determine cortisol concentrations in humans, pigs, dogs and rhesus hair [11,21,22,23] and in the same laboratory where the current samples were analysed [13]. The cortisol levels in each sample were replicated in two wells, and the reported values correspond to the mean of the two data obtained.

### 2.4. Complete Blood Count Analysis

The complete blood count (CBC) was analysed by an Auto Haematology Analyser BC-2800VetVR (Mindray, Shenzhen, China). This included the following haematological parameters:

White Blood Cells (WBC), Erythrocyte (RBC), Haemoglobin (HGB), Haematocrit (HCT), Mean Corpuscolar Volume (MCV), Mean Corpuscular Haemoglobin (MCH) and Mean Corpuscular Haemoglobin Concentration (MCHC).

### 2.5. Statistical Analysis

The results of the milk yield, milk composition, hair cortisol and blood count were analysed using ANOVA for repeated measurements, considering the sampling times (1, 6 and 10 months of lactation), the delivery order (primiparous, secondiparous and pluriparous) and the season at the time of sampling (autumn–winter, spring–summer) as fixed effects, and the subject as a random effect. The least significant means were compared by the *t*-test. Significant differences were considered at *p* < 0.05. Statistical analysis was carried out using the JMP software [24].

## 3. Results and Discussion

Table 1 shows that, at six months of lactation, there was a significant reduction in hair cortisol levels (*p* ≤ 0.01), which remained stable in the last samplings (10 months). 

The measuments of cortisol in hair showed a low coefficient of variation (mean = 3.9%; min = 0.2%; max = 11.1%). As the measured hair cortisol was assumed to reflect the hormone levels during the two months preceding the sampling, the values obtained for the first hair collection refer to the circulating cortisol levels during the months preceding and following parturition. Based on the higher cortisol found in the first samples, it was speculated that the delivery, as well as the beginning of lactation, represent stressful events for the jenny, thus increasing the levels of circulating and hair cortisol. This hypothesis is supported by the fact that in horses, the peri-parturition period is known to be characterized by an increase in cortisol [25]. The reduction and stabilisation of cortisol observed in the last samplings suggest that the jennies were temporarily stressed in the peri-parturition period but not in thereafter.

The donkey milk composition was within the ranges reported by Martini et al. [26], while the average milk production in the three considered periods was similar to that reported for the same breed by Licitra et al. [27]. The daily milk production was significantly lower (*p* ≤ 0.01) at the 10th month of lactation, while the protein content decreased starting from the 6th month (*p* ≤ 0.01). The urea milk content also showed a progressive increase during lactation (*p* ≤ 0.01).

These results are in line with previous studies for the same breed [16,28], the Ragusano donkey [29] and the Martina Franca donkey [30]. The somatic cell count was similar to that reported by Ragona et al. [31], with higher values at the 10th month of lactation (*p* ≤ 0.01). This trend in the final phase of lactation, associated with the simultaneous decrease in milk yield, may indicate a physiological involution of the mammary gland, as also reported by Salari et al. [16] and Pilla et al. [32].

The blood parameters were slightly lower than those detected in the same breed by Salari et al. [16], but within the range reported by Mori et al. [14] in Italian and Brazilian donkeys and by Lizarraga et al. [17]. During lactation, there was no significant difference in blood parameters except for the Mean Corpuscular Haemoglobin Concentration (MCHC g/dL), which was significantly lower at 10 months (*p* ≤ 0.01). A significant reduction in MCHC is usually an indication of an iron deficiency leading to hypochromic anaemia [33]. However, the lower MCHC in the last phase of lactation is in agreement with Salari et al. [16], who hypothesized that these variations, as they were not accompanied by changes in the MCV and the HGB content, are non-pathological and likely related to para-physiological changes during lactation.

The influence of parity on the quantity and quality of milk has been widely demonstrated in cows, especially primiparous cows which tend to produce less milk. However, little is known about donkeys used for milk production [34,35].

In the present study (Table 2), no significant differences were observed in hair cortisol based on parity. Even in primiparas, when higher values were expected because the first birth can involve significant stress, this increase was not evident.

No statistically significant differences were found in the milk yield according to parity; although, at first delivery, the jennies tended to produce slightly lower milk quantities.

On the other hand, the protein and casein content tended to decrease significantly and progressively (*p* ≤ 0.01) as the number of deliveries increased. The fat content showed an opposite trend and was significantly lower in primiparous jennies (*p* ≤ 0.05). This finding could be due to the lower milkability and less milk release of the primiparous jennies, which have not yet adapted to the milking routine, with a consequent increase in residual milk in the udder, which is known to be richer in fat. Similar results regarding the qualitative variability of donkey milk in relation to parity were also reported by Marchis et al. [35].

The reduced casein content in the pluriparous jennies could be used to feed human babies affected by cow’s milk protein allergy, as casein is considered to be one of the most important milk allergens [36].

With regards to the effect of parity on blood parameters, significantly lower WBC was found in pluriparous jennies (*p* ≤ 0.05), while significantly higher RBC was found in primiparous (*p* ≤ 0.05). Some studies [37,38] have reported higher RBC values in younger donkeys (foals); thus, higher RBC values in primiparous donkeys are probably due to the fact they are still growing. In addition, since both RBCs and WBCs tend to be lower in pluriparous jennies, it is possible that once the growth is complete, there is a physiological stasis of the regenerative capacity of bone marrow. Furthermore, the lower RBC values in adult animals seem to be balanced by an increase in erythrocyte size, as shown by the higher MCV values in pluriparous (*p* < 0.01) donkeys. This was confirmed by the fact that the haematocrit (HCT) did not differ between the groups, as also described by Dezutto et al. [15].

Table 3 shows the effects of the sampling season on hair cortisol, milk production and blood count. Thermal stress in dairy animals negatively affects milk yield and quality [39]. However, there is little information on the effects of thermal stress on donkeys [38,40], and these studies are not totally comparable because they were carried out at different latitudes, with different climatic conditions and on different breeds.

In this study, no significant differences were found in hair cortisol. However, a higher milk production (*p* < 0.05) was detected in spring–summer, which was associated with significantly lower casein and lactose values (*p* < 0.01). These results are probably due to a dilution effect linked to higher production. Similar trends for milk production, casein and lactose were also found in the same breed [28]. The urea milk content was also significantly higher in the spring–summer (*p* < 0.01).

These results seem to confirm the good adaptability of the Amiata donkey breed to both warm and temperate climates. In fact, some features of the Amiata suggest that it descends directly from the African donkey [41]. 

In general, seasonal changes in environmental temperature, relative humidity and air velocity influence the physiological responses of farm animals. Changes in the blood physiological parameters of the donkeys above the normal range indicate that the animals are stressed [42]. In addition, changes in haematological values, such as packed cell volume (PCV), RBC, MCV and MCHC, are used in determining the adaptation of animals to the environment [43,44]. 

In our study, significant differences in the MCH and MCHC were found during the two periods considered. The MCH and MCHC values were significantly lower in the spring–summer, as also reported by Zakari et al. [38]. Such changes could represent an adaptive response; however, more research is needed on the donkey species until the limits of the physiological ranges have been fully determined, especially considering the differences due to breed, age, sex, climatic conditions, management, etc.

## 4. Conclusions

In this study, donkey hair cortisol was evaluated for the first time. Hair cortisol levels were stable in terms of parity and during the productive seasons, but not during the lactation phase. In fact, higher hair cortisol values were found in the first hair sampling. This suggests that the jennies were able to deal with an increase in the blood cortisol level in the peripartum–early lactation period, as a result of the temporary stress during peri-parturition.

In addition, during the lactation phase, changes in the milk yield and quality, and changes in MCHC were highlighted as likely related to the physiological and para-physiological changes occurring during lactation.

The birth order influenced the milk fat, protein and casein contents. On the other hand, a decrease in the number of blood cells was found in secondiparous and pluriparous jennies compared to primiparous donkeys.

The production season affected some milk quality and blood parameters, such as MCH and MCHC, although the latter might represent the adaptation of the animals to environmental conditions. 

In conclusion, we believe that the results of this study contribute to a better understanding of the biochemical processes occurring in lactating jennies, and to the physiological and wellbeing of dairy jennies.

## Figures and Tables

**Table 1:** Effects of the lactation phase on hair cortisol, milk production and blood count.

	1 Month	6 Months	10 Months	*p*	RMSE
Mane hair					
HCCs (pg/mg)	11.74A	8.66B	9.37B	0.010	3.802
Milk yield and composition					
Milk yield/day (mL)	1535.01A	1337.67A	868.91B	0.001	425.418
Fat (%)	0.55	0.39	0.41	0.354	0.426
Protein (%)	1.57A	1.44B	1.41B	0.001	0.128
Casein (%)	0.88	0.83	0.82	0.060	0.089
Lactose (%)	6.85	6.88	6.87	0.790	0.133
Dry Matter (%)	9.39	9.32	9.45	0.728	0.520
SCC (number ×1000)	8.67AB	3.73B	12.80A	0.007	6.739
Urea (mg/mL)	20.74C	37.63B	43.82A	0.001	5.990
Blood analysis					
WBC (number × 10^9^)	13.63	13.26	11.59	0.122	3.771
RBC (number × 10^12^)	6.83	7.91	7.41	0.159	1.578
HGB (g/dL)	13.17	15.36	14.21	0.227	3.558
HCT (%)	40.72	48.62	45.08	0.082	9.887
MCV (fl)	60.50	60.95	61.10	0.790	2.982
MCH (pg)	19.56	19.72	19.10	0.095	1.021
MCHC (g/dL)	32.45A	32.38A	31.32B	≤0.001	0.917

SCC = Somatic Cell Count; WBC = white blood cell count; RBC = erythrocyte count; HGB = haemoglobin concentration; HCT = haematocrit; MCV = mean corpuscular volume; MCH = mean corpuscular haemoglobin; MCHC = mean corpuscular haemoglobin concentration. A–C Values within a row with different superscripts differing significantly at *p* < 0.01.

**Table 2:** Effect of parity on hair cortisol, milk production and blood count.

	Primiparous	Secondiparous	Pluriparous	*p*	RMSE
Mane hair					
HCCs (pg/mg)	8.97	10.04	10.76	0.169	3.802
Milk yield and composition					
Milk yield/day (mL)	1213.02	1348.29	1281.17	0.468	425.418
Fat (%)	0.16b	0.61a	0.58ab	0.015	0.426
Protein (%)	1.64A	1.51B	1.27C	0.003	0.128
Casein (%)	1.01A	0.90B	0.63C	0.001	0.089
Lactose (%)	6.86	6.86	6.88	0.665	0.133
Dry Matter (%)	9.29	9.60	9.27	0.013	0.520
SCC (number × 1000)	10.31	7.44	7.45	0.265	6.739
Urea (mg/mL)	31.35	37.15	33.68	0.249	5.990
Blood analysis					
WBC (number × 10^9^)	12.63ab	14.38a	11.49b	0.013	3.771
RBC (number × 10^12^)	8.10a	7.04b	7.00b	0.022	1.578
Hgb (g/dL)	15.48a	12.86b	13.41b	0.023	3.558
HCT (%)	47.79	42.14	44.50	0.114	9.887
MCV (fl)	59.05B	60.53B	62.97A	≤0.001	2.982
MCH (pg)	19.03	19.21	20.14	0.784	1.021
MCHC (g/dL)	32.26	31.81	32.09	0.475	0.917

SCC = Somatic Cell Count; WBC = white blood cell count; RBC= erythrocyte count; Hgb = haemoglobin concentration; HCT = haematocrit; MCV = mean corpuscular volume; MCH = mean corpuscular haemoglobin; MCHC = mean corpuscular haemoglobin concentration. a, b Values within a row with different superscripts differ significantly at *p* < 0.05. A–C Values within a row with different superscripts differ significantly at *p* < 0.01.

**Table 3:** Effects of the production season on hair cortisol, milk production and blood count.

	Spring-Summer	Autumn-Winter	*p*	RMSE
Mane hair				
HCCs (pg/mg)	9.29	10.08	0.437	3.802
Milk yield and composition				
Milk yield/day (mL)	1411.62a	1188.46b	0.049	425.418
Fat (%)	0.33	0.54	0.067	0.426
Protein (%)	1.51	1.46	0.181	0.128
Casein (%)	0.80B	0.88A	0.001	0.089
Lactose (%)	6.79B	6.90A	0.003	0.133
Dry Matter (%)	9.26	9.47	0.128	0.520
SCC (number × 1000)	7.56	10.20	0.397	6.739
Urea (mg/mL)	38.95A	30.89B	0.001	5.990
Blood analysis				
WBC (number × 10^9^)	12.41	13.29	0.465	3.771
RBC (number × 10^12^)	7.49	7.28	0.655	1.578
Hgb (g/dL)	14.25	14.25	0.998	3.558
HCT (%)	45.74	43.87	0.534	9.887
MCV (fl)	60.82	60.95	0.948	2.982
MCH (pg)	18.92B	20.01A	≤0.001	1.021
MCHC (g/dL)	31.17B	32.93A	≤0.001	0.917

SCC = Somatic Cell Count; WBC = white blood cell count; RBC = erythrocyte count; Hgb = haemoglobin concentration; HCT = haematocrit; MCV = mean corpuscular volume; MCH = mean corpuscular haemoglobin; MCHC = mean corpuscular haemoglobin concentration. a, b Values within a row with different superscripts differ significantly at *p* < 0.05. A, B Values within a row with different superscripts differ significantly at *p* < 0.01.

## Data Availability

The data presented in this study are available on request from the corresponding author.
